# An insert with less than spherical medial conformity causes a loss of passive internal rotation after calipered kinematically aligned TKA

**DOI:** 10.1007/s00402-021-04054-0

**Published:** 2021-07-15

**Authors:** Alexander J. Nedopil, Adithya Shekhar, Stephen M. Howell, Maury L. Hull

**Affiliations:** 1 Department of Orthopaedic Surgery, König-Ludwig-Haus, University of Würzburg, Würzburg, Germany; 2 California Northstate University, Elk Grove, USA; 3 Department of Biomedical Engineering, University of California, Davis, 451 E. Health Sciences Drive, Room 2303, Davis, CA 95616 USA

**Keywords:** Medial stabilized, Spherical, Conforming, Insert, Rotation, Total knee arthroplasty, Total knee replacement, Kinematic alignment, Calipered

## Abstract

**Introduction:**

In total knee arthroplasty (TKA), the level of conformity, a medial stabilized (MS) implant, needs to restore native (i.e., healthy) knee kinematics without over-tensioning the flexion space when the surgeon chooses to retain the posterior cruciate ligament (PCL) is unknown. Whether an insert with a medial ball-in-socket conformity and lateral flat surface like the native knee or a less than spherical medial conformity restores higher and closer to native internal tibial rotation without anterior lift-off, an over-tension indicator, when implanted with calipered kinematic alignment (KA), is unknown.

**Methods and materials:**

Two surgeons treated 21 patients with calipered KA and a PCL retaining MS implant. Validated verification checks that restore native tibial compartment forces in passive flexion without release of healthy ligaments were used to select the optimal insert thickness. A goniometer etched onto trial inserts with the ball-in-socket and the less than spherical medial conformity measured the tibial rotation relative to the femoral component at extension and 90° and 120° flexion. The surgeon recorded the incidence of anterior lift-off of the insert.

**Results:**

The insert with the medial ball-in-socket and lateral flat surface restored more internal tibial rotation than the one with less than spherical medial conformity, with mean values of 19° vs. 17° from extension to 90° flexion (*p* < 0.01), and 23° vs. 20°–120° flexion (*p* < 0.002), respectively. There was no anterior lift-off of the insert at 90° and 120° flexion.

**Conclusion:**

An MS insert with a medial ball-in-socket and lateral flat surface that matches the native knee’s spherical conformity restores native tibial internal rotation when implanted with calipered KA and PCL retention without over-tensioning the flexion space.

## Introduction

**Fig. 1:**
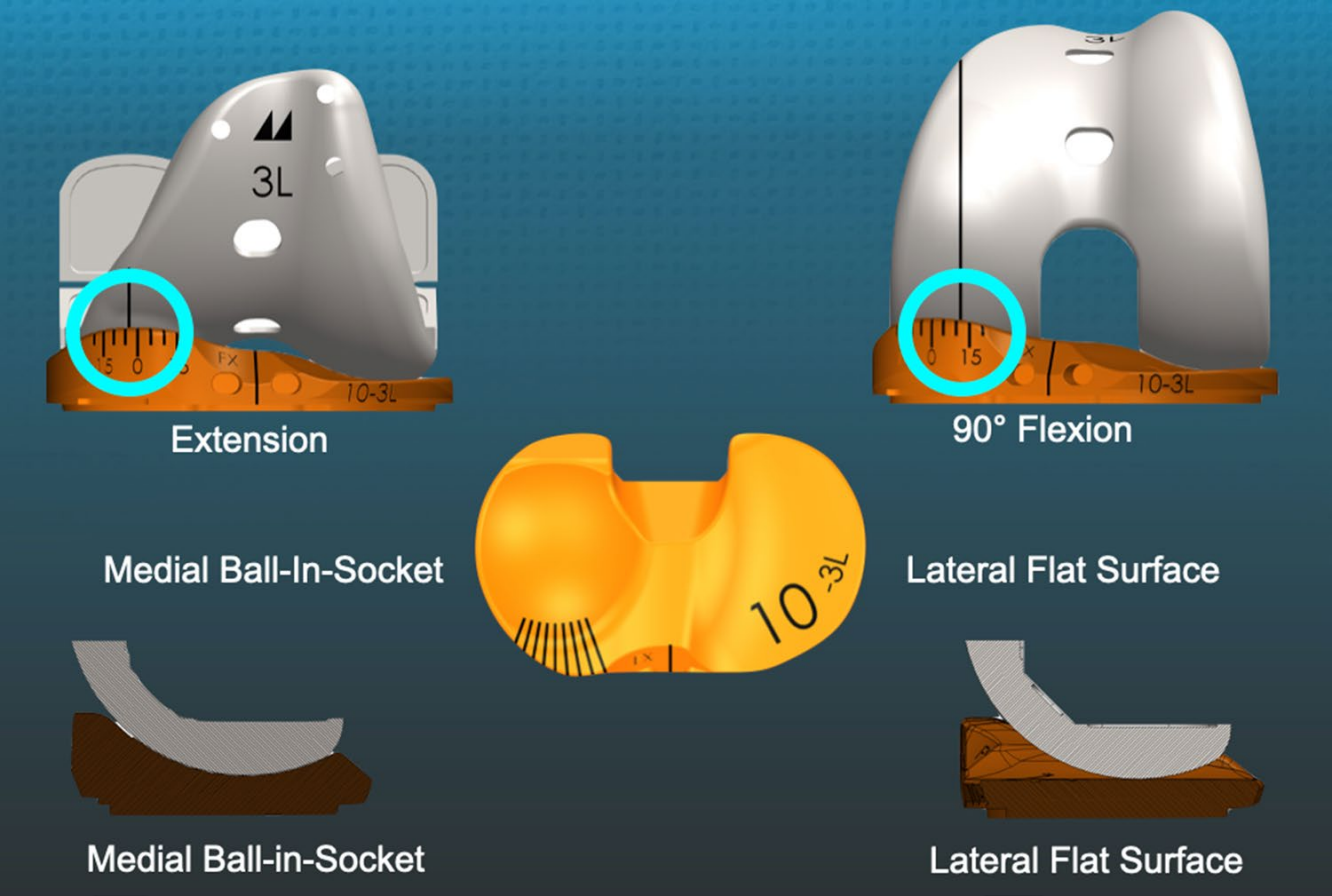
Schematics show an MS implant with a medial spherical femoral condyle and the different insert conformity between the medial ball-in-socket and lateral flat surface, and the anteromedial goniometer scale that measured the I–E orientation of the tibia relative to a longitudinal line on the trial femoral component with the TKA in 90° flexion

In total knee arthroplasty (TKA), the optimal level of conformity between the femoral component and tibial insert when the posterior cruciate ligament (PCL) is excised or retained is unknown. Comparisons of a medial stabilized (MS) design with PCL excision against PCL retaining (CR), posterior stabilized (PS), and ultra-congruent (UC) geometries showed that an MS insert with a medial ball-in-socket and a lateral flat surface with spherical conformity like the native knee restored greater medial anterior–posterior (A-P) stability and internal tibial rotation during gait than the others (Fig. 1). The lateral flat surface enabled internal tibial rotation, whereas the posterolateral rim of the PS, CR, and UCs’ insert stopped internal rotation like a chock block [1, 2]. Hence, articular geometry is a critical factor in governing knee kinematics [1].

Restoring the flexion and extension and internal tibial rotation of the native (i.e., healthy) knee with knee flexion are useful functional goals after TKA [3]. In the native knee, an intact PCL enables internal tibial rotation with the kinematic benefit of decreasing the Q-angle during knee flexion [4–6]. Decreasing the Q-angle optimizes the retinacular ligaments’ tension that guides patellofemoral tracking, which might reduce the risks of patellar tilt and lateral displacement and anterior knee pain in TKA [7, 8].

To assess whether an MS implant with PCL retention restores internal tibial rotation with passive knee flexion, it is of interest to evaluate a highly congruent medial conforming insert which constrains anterior–posterior (A–P) movement of the medial femoral condyle similar to the spherical medial compartment of the native knee as described by Freeman and Pinskerova [9]. Their knee dissections and image analysis showed that the medial femoral condyle hardly moves anterior–posterior from 0° to 120°, behaving like a ball-in-socket joint. The lateral tibia’s flat cartilage surface and posteriorly mobile lateral meniscus enable the lateral tibia's anterior movement about a longitudinal axis centered in the medial compartment. They reported 18° and 23° of internal tibial rotation from extension to 90° and 120° flexion, desirable arcs of tibial rotation for TKA that a trial insert goniometer can measure (Fig. 1) [4, 9, 10].

**Fig. 2:**
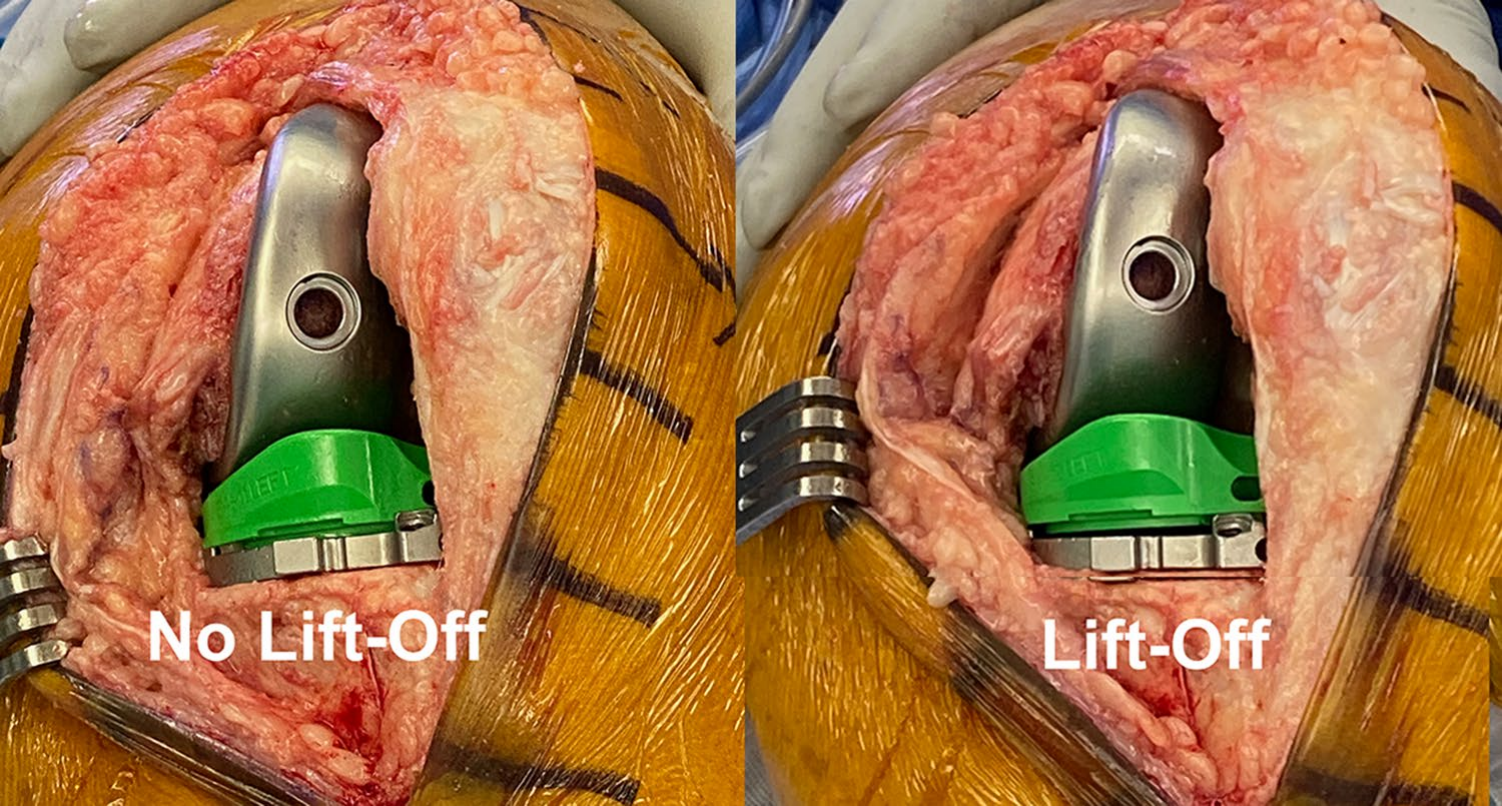
Intraoperative photographs of a left TKA in 90° flexion show no lift-off and lift-off of the trial insert (not goniometric) from the trial baseplate, which indicates kinematic conflict and PCL over-tension. The lift-off occurred between the insert and the trial baseplate because the insert does not lock into the baseplate and a cruciate stem firmly fixes the baseplate to the tibia

**Fig. 3:**
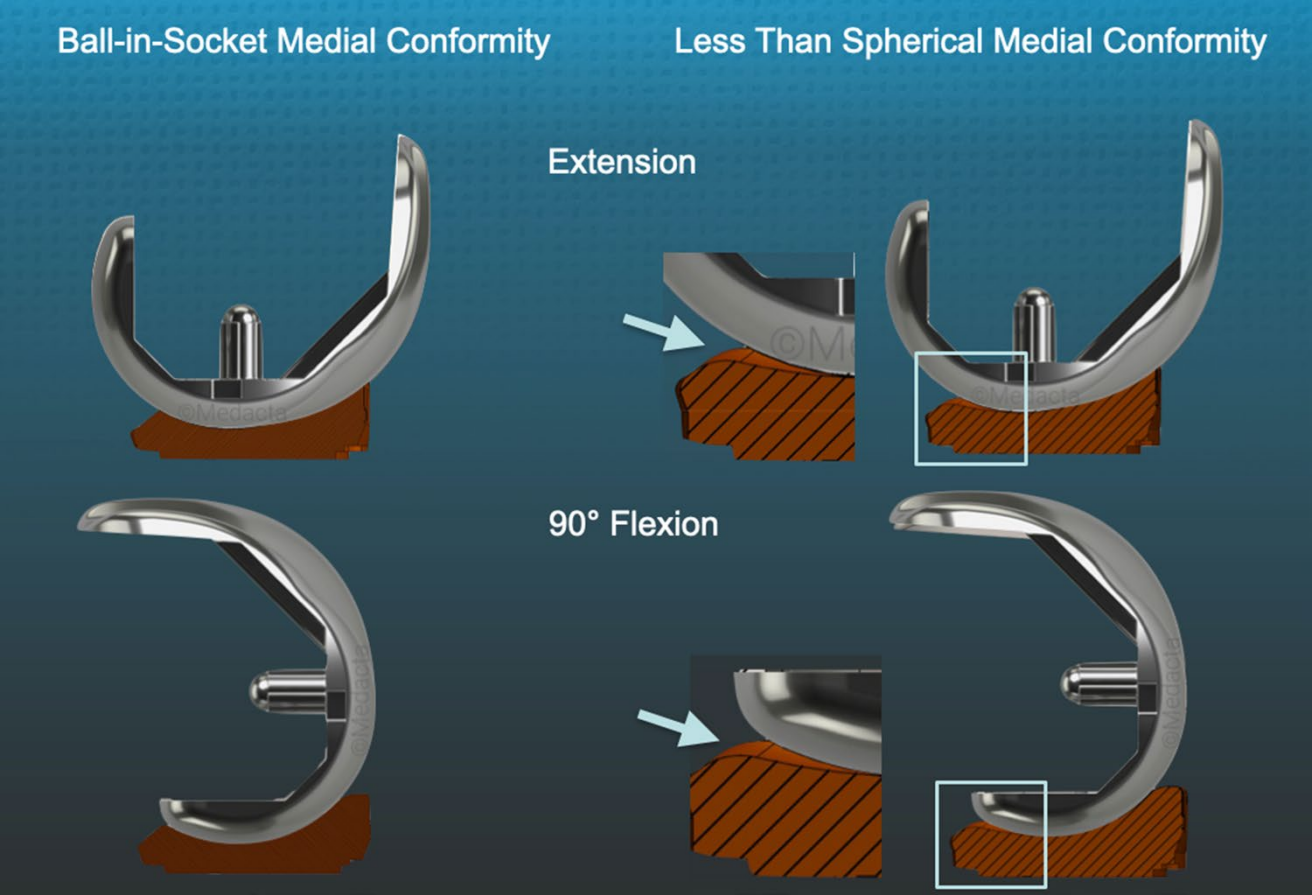
Schematics of a medial-view sectioned in the middle of the spherical femoral condyle show the difference in insert conformity with the knee in extension and 90° flexion. The arrows point to expanded sections (square) of the posterior region of the insert of the less than spherical conformity that shows loss of congruency, enabling abnormal A–P tibial motion and a loss of internal tibial rotation with flexion

Not only is implant design a critical factor governing knee kinematics, but also implant alignment is a critical factor as well [11]. As an alternative to mechanical alignment TKA with a patient reported dissatisfaction rate as high as 20%, kinematic alignment (KA) was conceived in 2006 to improve outcomes by restoring native knee kinematics without the release of healthy ligaments and without increasing the risk of revision surgery [12]. KA with PCL retention optimizes soft-tissue balance by restoring the patient’s pre-arthritic joint lines and native tibial compartment forces and laxities in passive flexion, which is evidence of not over-tensioning the PCL [13–16]. In TKA, anterior lift-off or ‘booking’ of the trial insert (or baseplate) detects an over-tensioned PCL and a tight flexion space [17] (Fig. 2). Because the optimal medial insert congruency for an MS design when used with calipered KA and PCL retention is unknown, there is a need to evaluate inserts with a ball-in-socket and a less than spherical medial conformity, which are contrasted schematically in the sagittal plane in Fig. 3.

The present study evaluated 21 patients with calipered KA and determined which medial insert conformity achieved higher internal tibial rotation at 90° and 120° flexion and whether one conformity more closely restored the values of internal tibial rotation reported for the native knee without evidence of an over-tensioned PCL and flexion space as detected by anterior lift-off.

## Methods and materials

Our institutional review board approved the study (IRB 1632230-1). Between mid-May 2020 and early June 2020, two surgeons treated 36 consecutive patients with a primary TKA using calipered KA, PCL retention, and patella resurfacing through a mid-vastus approach. Each patient fulfilled the Centers for Medicare and Medicaid Services guidelines for medical necessity for TKA treatment including: (1) radiographic evidence of Kellgren–Lawrence Grade II to IV arthritic change or osteonecrosis; (2) any severity of clinical varus or valgus deformity); (3) and any severity of flexion contracture. Patients were treated with a calipered KA TKA performed with PCL retention (GMK Sphere, Medacta International, Castel San Pietro, Switzerland) (Fig. 1). An implant company manufactured pairs of 3-D printed one-time use trial goniometers with either a spherical or less than spherical medial ball-in-socket with a lateral flat insert in 10, 11, and 12 mm thicknesses for size 3, 4, and 5 left and right tibial baseplates. Figure 3 schematically contrasts the sagittal difference in conformity between the between the spherical and less than spherical medial ball-in-socket conformity. Because of the goniometer insert’s limited inventory, the surgeons had to perform 36 consecutive primary TKAs before they could perform the analysis on 21 patients because of the limited inventory of five size 3, nine size 4, and seven size 5 trial goniometer inserts. The tibial baseplate has an anatomically shaped footprint and a posterior cut-out for retention of the PCL that, when best-fit to the tibial resection, sets the anterior–posterior (A-P) orientation parallel to the flexion–extension (F–E) plane of the pre-arthritic knee [18].

The sample size calculation used a 3° difference to detect in internal tibial rotation from 0° to 90° of flexion between the levels of spherical conformity of the medial insert. Assuming a Type I error (alpha) of 0.05, a power (1-beta) of 80%, and a standard deviation of ± 6°, the sample size was 18 patients.

Descriptive statistics of preoperative clinical characteristics, knee conditions, and function of included (*n* = 21) and not-included (*n* = 15) patients are shown (Table 1). Preoperatively, there were no significant differences in age, proportion of women, body mass index, extension, flexion, varus or valgus deformities, Oxford Knee Score, Knee Society Score, or Knee Function Score between included and not-included patients, which reduced the risk of a selection bias that could limit the generalization of the study’s findings.

**Table 1:** Preoperative patient demographics and clinical and radiographic characteristics of included and not-included patients

Preoperative demographics and clinical and radiographic characteristics	Included patients *N* = 21	Not-included patients *N* = 15	Significance
	Demographics		
Age (years)	70 (± 7.9)	68 (± 8.8)	n.s
Sex (male)	8 (38%)	7 (47%)	n.s
Body Mass Index (kg/m^2^)	29.2 (± 5.3)	30.2 (± 4.4)	n.s
	Preoperative motion, deformity, ACL condition, and Kellgren–Lawrence Score		
Extension (°)	7 (± 5)	7 (± 8)	n.s
Flexion (°)	112 (± 6.4)	110 (± 8.7)	n.s
Varus (+)/Valgus (−) Deformity (degrees)	− 12.2 (± 3.1)	− 10.8 (± 3.1)	n.s
Kellgren–Lawrence Score	3.6 (± 0.6)	3.4 (± 0.5)	n.s
	Preoperative function		
Oxford Score (48 is best, 0 is worst)	21 (± 8.4)	16 (± 6.5)	n.s
Knee Society Score	38 (± 11.7)	38 (± 16.4)	n.s
Knee Function Score	55 (± 21.5)	46 (± 16.1)	n.s

### Overview of the unrestricted calipered KA technique and accuracy analysis of component placement

For the femoral component, the varus–valgus (V–V) and I–E orientations and the A–P and proximal–distal (P–D) positions were set coincident with the patient’s pre-arthritic distal and posterior joint lines [13]. An accuracy analysis showed these steps restore the distal lateral femoral joint line of 97% of patients within the normal left to right symmetry and set the I–E orientation of the femoral component with a deviation of 0.3° (external) ± 1.1° from the KA target of the F–E plane of the patient’s knee [13, 14, 19, 20].

**Fig. 4:**
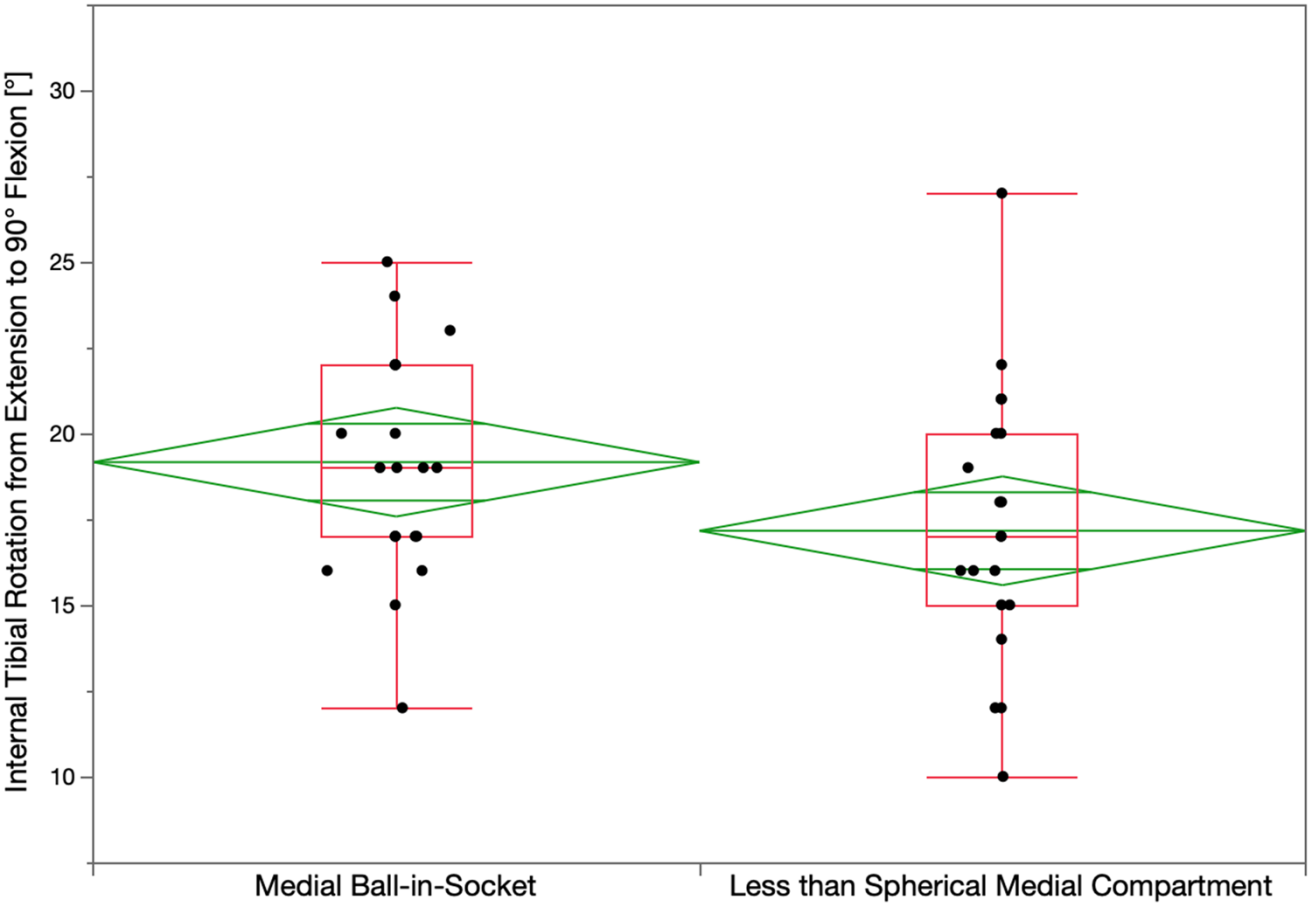
Box plots show that the mean internal tibial rotation from extension to 90° flexion (transverse line in the middle of the green diamond) of 19° for the ball-in-socket was significantly greater than the 17° for the less than spherical medial insert when implanted with calipered KA and PCL retention (*p* < 0.01). The top and bottom edges of the green diamond indicate the 95% confidence interval limits

**Fig. 5:**
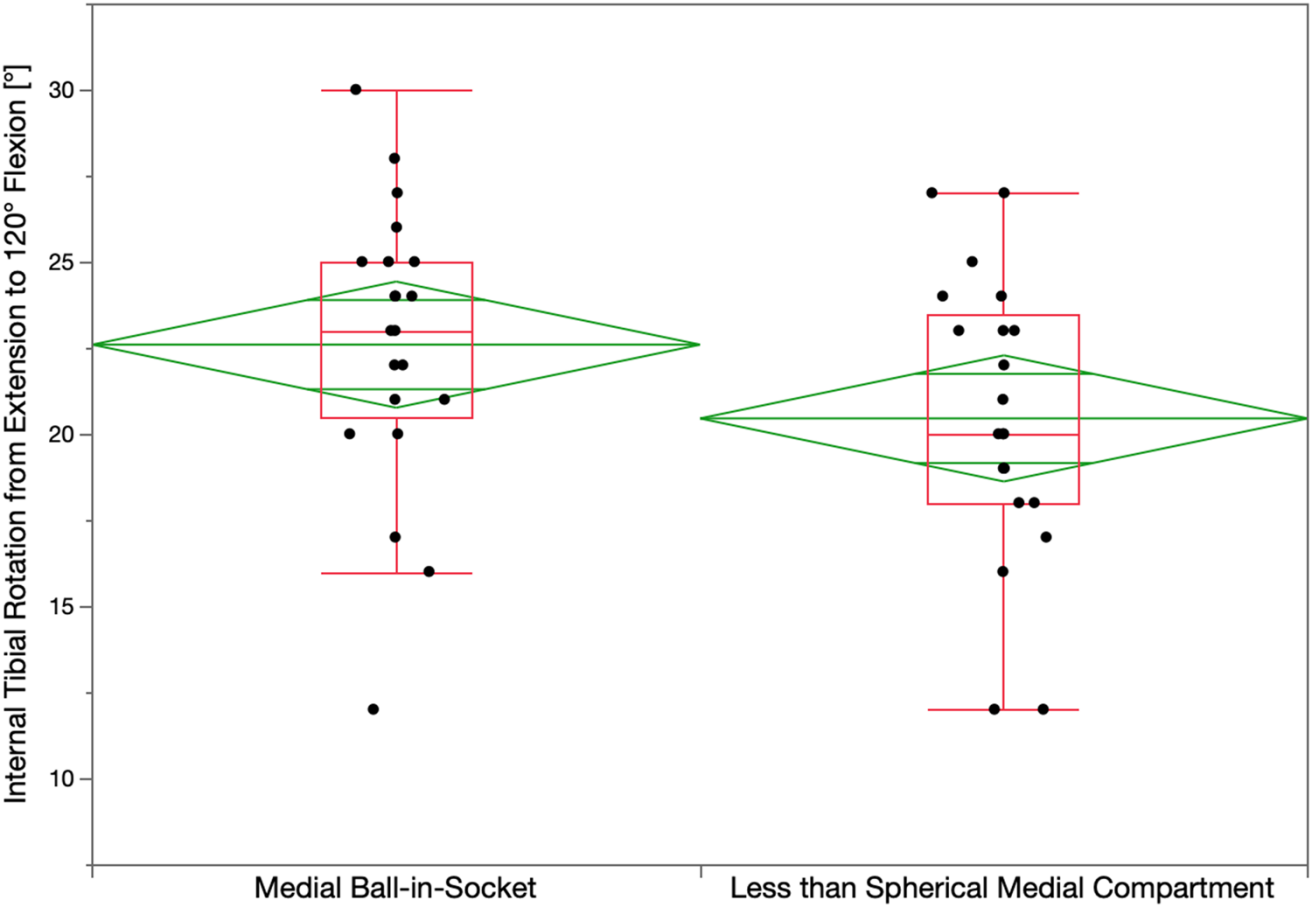
Box plots show that the mean internal tibial rotation from extension to 120° flexion of 23° for the ball-in-socket was significantly greater than the 20° for the less than spherical medial insert (*p* < 0.002)

The surgeon followed six options in a decision-tree to set the V–V and posterior slope orientation of the tibial component to restore the patient’s pre-arthritic tibial joint line and limb alignment and balance the knee by restoring the native tibial compartment forces (Figs. 4 and 5) [16, 21, 22]. The thickness of the resected tibial bone was measured using a caliper and the varus–valgus orientation of the proximal tibial resection was adjusted working in 1° or 2° increments by shaving the bone with a saw or using a varus or valgus tibial recut guide [23]. With the knee in extension, the tibial resection’s varus–valgus orientation was correct when the spacer block indicated a tight rectangular space, and there was little medial and lateral lift-off of the trial tibial insert from the femoral component during a varus–valgus laxity. An accuracy analysis showed these steps restore the proximal medial tibial joint line of 97% of patients within the normal left to right symmetry [14, 20, 24]. The posterior slope was adjusted by setting an angel wing inserted through the medial slot of the tibial guide parallel to the patient’s pre-arthritic slope. An accuracy analysis showed a 0° mean difference between the tibial component’s posterior slope and the patient’s pre-arthritic posterior slope [24]. A best-fit of the largest anatomically shaped trial tibial baseplate inside the cortical rim of the proximal tibial resection method set the I–E orientation and A–P and medial–lateral (M–L) positions. An accuracy analysis showed a mean 2° (external) ± 5° deviation of the I–E orientation of the tibial component from the KA target of the F–E plane of the patient’s knee [13, 14, 16, 18, 22, 25].

The following steps determined the optimal insert thickness within a ± 1 mm target. Place the knee in 90° flexion and palpate the PCL to verify that it is intact. Insert a goniometric tibial insert that matches the thickness of the spacer block. Place the knee in extension and verify that the knee hyperextends a few degrees, like the pre-arthritic knee. When the knee has a flexion contracture, insert a thinner insert or release the posterior capsule. Verify that the V–V laxity is negligible in full extension and the lateral compartment has a 3–4 mm gap and the medial compartment a negligible gap with the knee in 15°–30° flexion. When necessary, fine-tune the V–V plane of the tibial resection. Place the knee in 90° flexion and determine whether passive I–E rotation of the tibia approximates ± 15° like the native knee [15].

### Methods for measuring the orientation of the tibia with the insert goniometer and recording anterior lift-off of the trial insert

The scrub tech randomly selected either the spherical or less than spherical medial ball-in-socket and lateral flat insert trial goniometric insert, which the surgeon inserted. The mid-vastus exposure maintained the resurfaced patella in the prosthetic trochlea throughout the motion arc. The surgeon used the back of the wrist to lift the heel and passively extend the knee without applying an I–E moment to the ankle. The surgeon recorded the angle in degrees where the reference line on the medial condyle of the trial femoral component intersected the goniometer’s angular arc (+ external/− internal) (Fig. 1). The surgeon also recorded the tibial orientation at 90° and 120° flexion and anterior lift-off of the insert with the foot resting on the operating table and supporting the leg’s weight. The surgeon repeated these assessments with the other insert.

### Statistical analysis

Data were analyzed using statistical software (JMP® Pro 15.2.1, SAS, Cary, NC, USA). The mean and standard deviation described the continuous variables. A Student’s paired *t *test determined the significance of differences in internal tibial rotation from extension to 90° and 120° flexion between the insert with the medial ball-in-socket and the less than spherical conformity. Significance was *p* < 0.05.

To quantify reproducibility, two observers (SMH and AJN) measured the I–E orientation of the tibia at maximum extension and 90° flexion in seven knees. A two-factor mixed-model analysis of variance (ANOVA) with random effects computed the intra-class correlation coefficient (ICC). The first factor was the observer (2 levels), and the second was the patient (7 patients). An ICC value of > 0.9 indicates excellent agreement, and 0.75–0.90 indicates good agreement. ICC values of 0.82 for the measurement of tibial orientation at extension and 0.87 at 90° flexion indicated good reproducibility.

## Results

The mean I–E tibial orientation in extension for the insert with the ball-in-socket and less than spherical medial conformity was comparable (*p* = 0.128), and the insert with the ball-in-socket had 2° and 3° more internal tibial orientation at 90° and 120° (*p* < 0.0001, *p* < 0.0001), respectively. The insert with the ball-in-socket restored more internal tibial rotation than the one with less than spherical medial conformity, with mean values of 19° ± 3° vs. 17° ± 4° from extension to 90° flexion (*p* < 0.01), and 23° ± 4° vs. 20° ± 4° degrees from extension to 120° flexion (*p* < 0.002), respectively (Figs. 4 and 5). There was no evidence of over-tensioning of the PCL and flexion space as neither medial insert conformity had anterior lift-off at 90° and 120°.

## Discussion

The most important finding of the present study of 21 patients treated with calipered KA and PCL retention was that an MS insert with a spherical medial ball-in-socket and lateral flat surface restored a modest 3° more internal tibial rotation than an insert with less than spherical medial conformity indicating that subtle differences on sagittal conformity affect passive knee kinematics. Because the internal tibial rotation with a spherical medial ball-in-socket insert was comparable to values reported for the native knee and there was no anterior lift-off, the calipered KA did not over-tension the PCL and flexion space.

The present study showed that an insert with a medial ball-in-socket and a lateral flat surface is an optimal MS design when implanted with calipered KA and PCL retention because it restored passive internal tibial rotation comparable to the values of 18° at 90° of flexion and 23° at 120° flexion reported for the native knee even with excision of the anterior cruciate ligament (ACL) which is an unexpected finding not previously reported for the TKA and the native knee [4, 9, 10]. In the native knee, an intact PCL and ACL enable I–E tibial rotation, and sectioning of these ligaments eliminates it [5]. An explanation for the TKA’s restoration of native internal tibial rotation is the insert’s medial ball-in-socket conformity provided a mechanical A–P stop like intact cruciate ligaments and preserved the native PCL tension that drives the rotational kinematics [4–6]. The absence of anterior lift-off of the insert indicated that over-tensioning of the PCL and flexion space did not occur at 90° and 120° flexion and is explained by calipered KA accurately setting the femoral and tibial components coincident within 0 ± 0.5 mm of the pre-arthritic joint lines without releasing healthy ligaments, which restores native tibial compartment forces and laxities during passive flexion [13–16, 19, 22, 26].

The present study showed that native internal tibial rotation is compromised when the insert’s medial compartment's conformity is less than spherical. Less than spherical medial insert conformity enables anterior tibial motion that slackens the PCL and lowers the ligament force that drives tibial rotation [1, 2]. The PCL’s resection in the cadaveric knee reduced internal tibial rotation at high-flexion angles beginning at 60° [6]. A 3-D fluoroscopic analysis of a deep knee bend in patients with a PCL injury in one knee and the other intact showed a decreased internal tibial rotation throughout the range of flexion in the PCL-deficient knee, which correlated with patellar tilt (*R*^2^ = 0.73) and medial–lateral patellar translation (*R*^2^ = 0.63) [27, 28]. Hence, surgeons and bioengineers should consider restoring the native knee’s kinematic coupling between internal tibial rotation and patellofemoral tracking and loading when developing surgical techniques such as TKA [28].

The present study has several limitations. Because the I–E measurement of tibial orientation provided by the insert goniometer is for a medial ball-in socket MS design, its usefulness for intraoperatively measuring tibial rotation needs to validated for shallower, non-spherical MS design that enable large amounts of A–P motion like the PCL retaining (CR), posterior stabilized (PS), and ultra-congruent (UC) geometries [1, 2]. The degree of internal tibial rotation measured in the present study at 90° and 120° might not apply to PS, PCL retaining, and ultra-congruent geometries with a posterolateral insert rim that functions as a chock block. The degree of internal tibial rotation is likely less for implants placed with mechanical alignment that does not restore the patient’s pre-arthritic joint lines, and ligaments are released to encourage motion in the over-constrained TKA [29, 30]. While the restoration of normal knee motion is the target of knee surgery, and the spherical medial conformity restored native internal tibial rotation that is associated with less post-operative pain, the present study did not determine whether the loss of 3° of internal tibial rotation from using a less than spherical medial conformity has any clinical adverse effects [31–34].

## Conclusion

Surgeons that use an MS implant should understand that the level of medial conformity determines the magnitude of internal tibial rotation and that a medial ball-in-socket and lateral flat insert that matches the conformity of the native knee restores native internal tibial rotation when implanted with calipered KA and PCL retention without over-tensioning the flexion space. In contrast, a less than spherical medial conformity causes a loss of internal tibial rotation.
